# Malaria Epidemiology and Control within the International Centers of Excellence for Malaria Research

**DOI:** 10.4269/ajtmh.15-0006

**Published:** 2015-09-02

**Authors:** William J. Moss, Grant Dorsey, Ivo Mueller, Miriam K. Laufer, Donald J. Krogstad, Joseph M. Vinetz, Mitchel Guzman, Angel M. Rosas-Aguirre, Socrates Herrera, Myriam Arevalo-Herrera, Laura Chery, Ashwani Kumar, Pradyumna K. Mohapatra, Lalitha Ramanathapuram, H. C. Srivastava, Liwang Cui, Guofa Zhou, Daniel M. Parker, Joaniter Nankabirwa, James W. Kazura

**Affiliations:** Department of Epidemiology, Johns Hopkins Bloomberg School of Public Health, Baltimore, Maryland; Department of Medicine, University of California San Francisco, San Francisco, California; Walter and Eliza Hall Institute, Melbourne, Australia; Center for Vaccine Development, University of Maryland School of Medicine, Baltimore, Maryland; Department of Tropical Medicine and the Center for Infectious Diseases, Tulane University, New Orleans, Louisiana; Department of Medicine, University of California San Diego, La Jolla, California; Alexander von Humboldt Institute of Tropical Medicine, Universidad Peruana Cayetano Heredia, Lima, Peru; Caucaseco Scientific Research Center, Cali, Colombia; Malaria Vaccine and Drug Development Center, Cali, Colombia; School of Health, Universidad del Valle, Cali, Colombia; Department of Chemistry, University of Washington, Seattle, Washington; National Institute of Malaria Research-Goa, Panaji, Goa, India; Regional Medical Research Centre-Northeast, Dibrugarh, Assam, India; Department of Biology, New York University, New York, New York; National Institute of Malaria Research-Nadiad, Gujarat, India; Department of Entomology, Pennsylvania State University, University Park, Pennsylvania; Program in Public Health, University of California at Irvine, Irvine, California; Department of Medicine, Makerere University College of Health Sciences, Kampala, Uganda; Center for Global Health and Diseases, Case Western Reserve University, Cleveland, Ohio

## Abstract

Understanding the epidemiological features and metrics of malaria in endemic populations is a key component to monitoring and quantifying the impact of current and past control efforts to inform future ones. The International Centers of Excellence for Malaria Research (ICEMR) has the opportunity to evaluate the impact of malaria control interventions across endemic regions that differ in the dominant *Plasmodium* species, mosquito vector species, resistance to antimalarial drugs and human genetic variants thought to confer protection from infection and clinical manifestations of plasmodia infection. ICEMR programs are conducting field studies at multiple sites with the aim of generating standardized surveillance data to improve the understanding of malaria transmission and to monitor and evaluate the impact of interventions to inform malaria control and elimination programs. In addition, these epidemiological studies provide a vast source of biological samples linked to clinical and environmental “meta-data” to support translational studies of interactions between the parasite, human host, and mosquito vector. Importantly, epidemiological studies at the ICEMR field sites are integrated with entomological studies, including the measurement of the entomological inoculation rate, human biting index, and insecticide resistance, as well as studies of parasite genetic diversity and antimalarial drug resistance.

## Introduction

Understanding the epidemiological features and metrics of malaria in endemic populations is a key component to monitoring and quantifying the impact of current and past control efforts to reduce morbidity due to plasmodia infection to a level that is acceptable from a public health perspective, eliminate malaria by decreasing the reproduction number (R_0_) to a level at which transmission is not sustained by local mosquito vectors in a defined geographic region, and ultimately to eradicate malaria by irrevocably reducing the global incidence of plasmodia infection to nil.^1^ Historically, the goal of the Global Malaria Eradication Program (GMEP) initiated by the World Health Organization (WHO) in 1955 was to eliminate malaria in all endemic areas of the world with the exception of Africa, where the goal was to control malaria. Implementation of the major interventions available at the time, specifically antimalarial drugs (primarily chloroquine) to reduce infection prevalence in human populations and dichlorodiphenyltrichloroethane (DDT) to shorten mosquito vector viability, resulted in the elimination of *Plasmodium falciparum* and *P. vivax* transmission in 37 countries where these infections were endemic in 1950.^2^ By the mid-1970s, malaria was eliminated in 27 countries of Europe and North America.^3^ The remarkable success of the GMEP in these areas was aligned with the improvement of the local public health infrastructure, the commitment of financial and human resources that were not realized until after the Second World War, and sustained political commitment. In contrast, in many other countries where preexisting transmission was high to moderate, efforts at elimination were largely abandoned by the late 1960s because of the unanticipated development of parasite resistance to chloroquine and decreasing efficacy of DDT against mosquito vectors.

The metrics used to monitor and evaluate changes in malaria transmission at the time varied among, and even within, regions and countries.^4^ Indicators commonly used for surveillance and monitoring included the prevalence of blood stage infection determined by the microscopic examination of blood smears, the estimated number of deaths attributed to malaria recorded by national or local health authorities, and the incidence of clinical malaria. Aside from infection diagnosed by microscopic inspection of blood smears, the sensitivity and specificity of metrics based on death and clinical manifestations attributable to plasmodia infection were unclear, particularly, in the many areas where other causes of acute febrile illness and anemia in children were common. Meaningful analyses and comparison of progress toward elimination across different endemic regions with varying malaria epidemiology was compromised given the lack of consensus on the use of a standardized and validated set of metrics.

In the wake of the abandonment of the GMEP, the prevalence of infection and malaria-related deaths increased in Africa and other areas where transmission was sustained. Because of the increasing number of children and pregnant women in Africa who experienced malaria morbidity and death during the past decades of the twentieth century, the WHO created the Roll Back Malaria program.^5^ Launched in 1998, the program was based primarily on the deployment of artemisinin combination therapy (ACT), distribution of insecticide-treated bed nets (ITNs) and to a lesser extent, indoor residual spraying (IRS) of insecticide.^6^ More recently, the changing epidemiology of *P. falciparum* malaria in Africa was assessed from 2000 to 2010, the time period over which these interventions were implemented.^7^ Using published and publically available data from throughout the region, spatial and temporal trends of *P. falciparum* infection were analyzed among 2- to 10-year-old children (PR*Pf*_2–10_) as an indicator of transmission. Downward trends in *P. falciparum* transmission were noted in many areas. However, a major conclusion of this comprehensive analysis was that 57% of African residents continued to live in areas where transmission was moderate to high in 2010.

What do these not-so-recent and recent reports of malaria epidemiology mean in the context of the National Institutes of Health International Centers of Excellence for Malaria Research (ICEMR) effort, now in the fourth year since its inception? The ICEMR program was created in a new era with a goal that goes beyond malaria “control,” an era in which eradication has been put back on the table as an achievable goal that might benefit the generation of children born in endemic areas today.^8^ Knowledge of the cell biology, genetics, genomics, and biochemistry of the parasite, particularly in the case of *P. falciparum* but less in *P. vivax* (the other major *Plasmodium* species important to human health), has advanced remarkably over the past several decades. Equally important, point-of-care diagnostic tests for blood stage infection (i.e., rapid diagnostic tests [RDTs]) and highly sensitive polymerase chain reaction assays that detect low-density blood stage infections that may sustain transmission of infection from humans to mosquitos^9^ are widely used. In addition, antimalarial regimens such as ACTs and wide deployment of ITNs appear to have reduced morbidity and allowed significant progress toward elimination in low transmission settings such as the Solomon Islands and Vanuatu.^10^ The ICEMR program thus has the opportunity to evaluate the impact of these and future interventions across endemic regions, which differ in the dominant *Plasmodium* species, mosquito vector species, resistance to antimalarial drugs, and human genetic variants thought to confer protection from infection and clinical manifestations of malaria. These evaluations can be conducted in greater depth than routinely done by national malaria control programs, including detailed molecular analyses of parasites and vectors and more sophisticated spatial analyses, albeit in more limited geographical areas.

There now exist not only historically used metrics to monitor and evaluate the impact of interventions to control and eliminate malaria, such as infection and malaria attributable disease prevalence and deaths, but also highly sensitive molecular and genetic tools that reflect subtle changes in the epidemiology and transmission dynamics of *P. falciparum* and *P. vivax* (Table 1 ). For example, the impact of key interventions such as population coverage with (ITNs), IRS, and case management can be mapped and tracked using newer metrics such as the force of infection^11^ (the number of new infections per person per unit time as determined by molecular genotyping to quantify exposure to new *Plasmodium* clones over time) and seroconversion rate (calculated by fitting a reverse catalytic model to age-specific prevalence of antibody responses to a single or defined set of recombinant malaria proteins expressed by various stage of the parasite lifecycle). The following sections describe how the ICEMR program represents a unique opportunity to compare the validity and utility of these and other newly developed metrics of transmission intensity and clinically relevant indicators that can be used in a standardized manner across the major malaria-endemic areas of the world to evaluate and guide malaria control and elimination strategies.

## ICEMR Field Studies of Malaria Surveillance, Monitoring, and Evaluation

Given the diverse and evolving nature of malaria transmission and its clinical manifestations, it is critical to generate basic epidemiological information across a range of settings. The ICEMR programs are uniquely positioned to capture this shifting epidemiology in real time across the globe. All ICEMR programs are conducting field studies at multiple sites with the aim of generating surveillance data to improve understanding of malaria transmission and to monitor and evaluate the impact of interventions to inform malaria control and elimination programs. In addition, these epidemiological studies provide a vast source of biological samples linked to clinical and environmental “meta-data” to support translational studies of interactions between the parasite, human host, and mosquito vector. The basic types of epidemiological studies being conducted by the ICEMR programs are described, with a focus on methodological issues, types of malaria indicators generated, and the strengths and weakness of each study design (Table 2).

### Health facility-based surveillance.

ICEMR field activities can be divided into four general categories: health facility-based surveillance, cohort studies, cross-sectional surveys, and entomological surveys. Health facility-based surveillance provides an efficient means of collecting basic epidemiological data on large numbers of patients presenting to health centers. These data are generally part of a country’s routine health management information systems and are one of the main sources of data reported to the WHO and Roll Back Malaria.^12^ Data collected from health facility-based surveillance include the number of symptomatic patients with suspected malaria, numbers of cases receiving a diagnostic test, the number of confirmed malaria cases, and the number of malaria deaths. Indicators used include the test positivity rate (the proportion of patients with suspected malaria who are tested for infection and test positive), malaria case incidence (i.e., confirmed malaria cases per 1,000 persons per year), and malaria mortality rate (inpatient malaria deaths per 100,000 persons per year). The primary advantage of health facility-based surveillance data is the relative ease of collecting longitudinal data on a large number of patients at different spatial scales, up to the national level. The primary disadvantages of health facility-based data is that it is often incomplete and of poor quality, limited by variation in health-seeking behaviors, and fails to capture asymptomatic and subpatent infections. Many patients may not present to the formal health-care system and even when they do, diagnostic testing for malaria is often not performed or is inaccurate. Indeed, malaria case detection rates captured through health facility-based surveillance systems are lowest in countries with the highest estimated burden of malaria.^12^ Caution should be taken in the interpretation of temporal trends in malaria metrics derived from health facility-based surveillance due to changes in health-seeking behavior; case definitions; the utilization, methods (i.e., microscopy versus RDT), availability and quality of laboratory diagnostic testing used; and the rates and accuracy of reporting. All of the ICEMR programs are conducting health facility-based surveillance at a range of outpatient and inpatient facilities, usually in collaboration with local governments. Advantages to some of these ICEMR-affiliated facilities are that additional resources are being applied to improve the quality and timeliness of data through standardized approaches to diagnostic testing, quality control measures, reliance on laboratory confirmed cases, and the use of electronic records and short message service text messages.

### Cohort studies.

Cohort studies are generally considered the gold standard for estimating the burden of malaria in a defined population. These studies involve following a group of study participants (ideally representative of the target population of interest) over time with a combination of active and passive surveillance activities to measure a variety of malaria indicators. Common indicators include malaria incidence, period prevalence of parasitemia and anemia, and force of infection (definitions provided in Table 1). The primary advantages of cohort studies are the high quality and breadth of data that can be generated. A well-conducted cohort study has the ability to accurately capture all malaria cases (both clinical and subclinical) and person-time at risk, necessary requirements for estimating incidence measures and temporal changes. Cohort studies can be used to estimate the impact of population level interventions and are a rich source of biological samples linked to a variety of other “meta-data.” The primary disadvantages of cohort studies are they are expensive and logistically challenging to conduct. In addition, data from cohorts may not be generalizable due to their sampling frame, changes in the composition of the cohort over time, and the fact that the cohort study itself may influence outcomes (for example, the need to treat all participants diagnosed with malaria and improved access to medical care). In low transmission settings, the incidence of malaria may be too low to justify the use of a cohort study design. Historically, many high-quality cohort studies, such as the Garki Project,^13^ were conducted in high transmission settings with fewer focusing on malaria across a range of epidemiological settings. The ICEMR programs are addressing this gap by conducting cohort studies over extended periods in numerous sites around the globe. These studies should provide a wealth of contemporary descriptive data, which will be strengthened by efforts to standardize methodologies and metrics, combine datasets, share biological samples for translational studies, and develop novel interactive data management tools for exploring these rich and complex datasets.

### Cross-sectional surveys.

Cross-sectional surveys provide another common source of data used for malaria surveillance and monitoring and evaluation. Cross-sectional surveys generally involve administration of a questionnaire and the collection of blood samples from members of households selected using probability sampling. Common indicators generated from cross-sectional surveys include coverage levels of preventive interventions (ITNs and IRS), fever case management practices, health-seeking behaviors, health status (under five mortality rate and anemia), and parasite prevalence for clinical and subclinical malaria (definitions provided in Table 1). Examples of national cross-sectional surveys include Demographic Health Surveys and Malaria Indicator Surveys. These data are commonly used by the WHO and Roll Back Malaria as well for malaria risk mapping, such as that done by the Malaria Atlas Project.^14^ Advantages of cross-sectional surveys are they are relatively easy to do, provide a large amount of information, and are generally representative of the population. However, they are relatively expensive and not performed frequently. Disadvantages of cross-sectional surveys include limited ability to capture data on malaria morbidity and monitor trends over shorter periods or on a fine spatial scale. All of the ICEMR programs are conducting cross-sectional surveys. Most of these surveys are being conducted repeatedly in defined geographic areas where other field activities are being carried out concurrently, such as entomological surveys. These approaches should improve our understanding of the relationships between indicators derived from different study designs and estimate the impact of changes in the coverage levels of control interventions with clinically relevant indicators measured longitudinally.

### Entomological surveys.

Entomological surveys comprise a broad range of methodologies aimed at generating data on the mosquito vector and its interaction with the human host. Methods commonly used to collect mosquitoes include human landing catches, Centers for Disease Control and Prevention light traps, pyrethrum spray catches, exit traps, household aspiration, screen barriers, and Shannon traps. Collection methods are based on the resting and biting behaviors of different *Anopheles* species. Once mosquitoes are collected, a variety of methods can be used for species identification, identifying sources of blood meals, and determining if the mosquito is infected with various stages of the malaria parasite. Commonly used indicators of transmission intensity include the human biting rate, the sporozoite rate, and the entomological inoculation rate (EIR). The advantages of entomological surveys are they can provide a rich source of data and the only direct measures of transmission. The primary disadvantage of entomological surveys is the lack of standardization in methods used to collect and interpret data, as well as the limitations of collection methods to capture mosquitoes feeding outdoors or during the day. Estimates of indicators derived from entomological surveys may be limited by precision and accuracy and lack the ability to discriminate small-scale spatial or temporal variability. This is especially true in low transmission settings where it may not be possible to collect sufficient numbers of mosquitoes or detect sporozoites. Entomology surveys are generally not done outside of the research setting due to logistical challenges and the need for specialized equipment, training, and expertise. All of the ICEMR programs are conducting some variation of entomological surveys. The methods used vary widely, largely because of differences in the local environments, characteristics of predominant vectors, and prior experience. Efforts are ongoing to standardize protocols, reagents, and analytical methods across the ICEMR programs. Information gained from these entomological surveys will fill a knowledge gap and improve the quality of these data so critical to improve our understanding of malaria transmission dynamics.

## Key Malaria Metrics at ICEMR Research Sites

**Figure 1.**
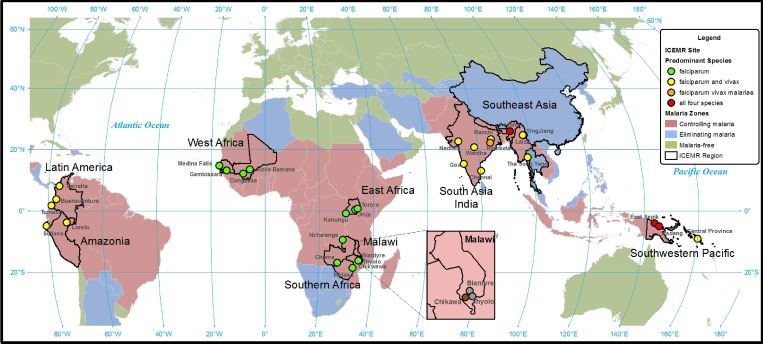
Predominant parasite species at the International Centers of Excellence for Malaria Research (ICEMR) research sites.

All four major parasite species infecting humans are represented in the ICEMR research sites (*Plasmodium knowlesii* is not a predominant parasite species at any ICEMR research site) (Table 3, Figure 1

). As expected, *Plasmodium falciparum* is the predominant parasite species at the 13 ICEMR research sites in Africa, with *P. vivax* and *P. falciparum* found throughout ICEMR research sites in South America and Asia. All four major parasite species are transmitted within ICEMR research sites in Papua New Guinea and southeast Asia.

**Figure 2.**
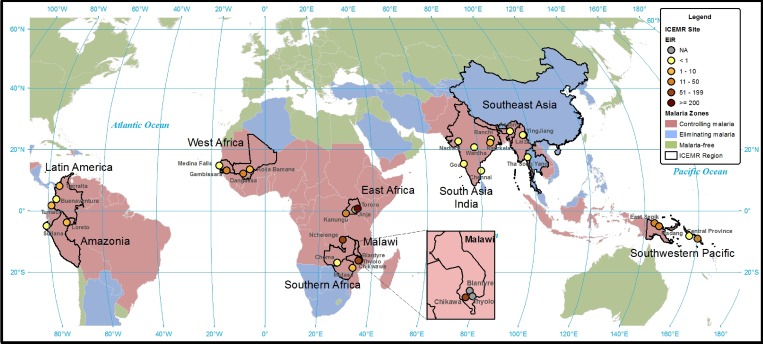
Approximate annual entomological inoculation rates at International Centers of Excellence for Malaria Research (ICEMR) research sites.

**Figure 3.**
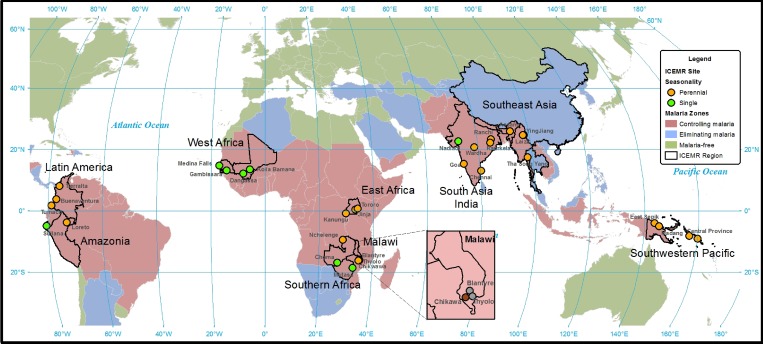
Seasonality of malaria transmission at the International Centers of Excellence for Malaria Research (ICEMR) research sites.

**Figure 4.**
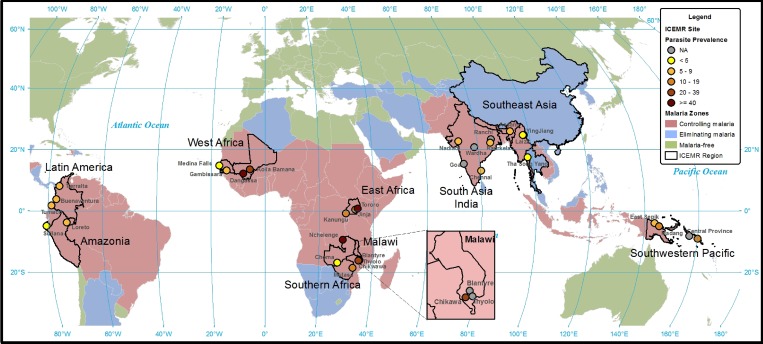
Approximate parasite prevalence at the International Centers of Excellence for Malaria Research (ICEMR) research sites.

Malaria transmission intensity as measured by the annual EIR varies widely across ICEMR research sites, ranging from below one infectious bite per year at sites in southeast Asia, Nadiad in India, Buenaventura in Colombia, Sullana in Peru, and Choma District in Zambia to 310 infectious bites per year in Tororo, Uganda (Table 3, Figure 2

). These differences reflect variation in major vector species, abundance, breeding habitats, and feeding behaviors as well as differences in the methods used to estimate EIR. Seasonal differences also determine malaria transmission intensity and vary across ICEMR sites (Table 3, Figure 3

). Both transmission intensity and seasonal patterns determine the interventions needed to achieve control or elimination. The parasite prevalence varies widely across ICEMR sites, from less than 1% in regions moving toward elimination to as high as 60% in Tororo, Uganda (Table 3, Figure 4

).

The ICEMR field sites reflect the range of commonly used malaria control interventions. Although the ICEMR investigators are not engaged in implementing malaria control interventions, the ICEMR field sites provide a platform to evaluate the effectiveness of current and future interventions implemented by national control programs. RDTs and artemisinin combination therapies are used for case management at many ICEMR sites. Malaria is confirmed by microscopy at ICEMR sites in Latin America and India. Intermittent presumptive treatment of malaria in pregnancy is used at ICEMR sites in sub-Saharan Africa and the southwest Pacific but not at sites in Latin America or India, where the combination of chloroquine and primaquine is used to treat disease due to *P. vivax* (with the exception of pregnant women). Reactive case detection, in which individuals residing within a define radius of a symptomatic index case are tested and treated for malaria, is conducted by the national malaria control programs in low transmission settings at ICEMR sites in Mali, Zambia, and southeast Asia that are moving toward elimination, as well as in Colombia for urban malaria elimination. For vector control, ITNs are distributed at almost all ICEMR sites but with varying levels of coverage (Table 3), and IRS is used at ICEMR sites in east and southern Africa and at some sites in India, southeast Asia, and southwest Pacific (Table 3).

## Discussion

The epidemiology of malaria is heterogeneous and highly focal, representing a diversity of parasites, vectors, seasonal patterns, and transmission intensities.^15^ Strategies for control and elimination must be adapted to the local epidemiological and entomological conditions. The ICEMR research sites represent this range of epidemiological diversity across tropical and subtropical regions in four continents. Even at smaller spatial scales, within countries, provinces and districts, the epidemiology of malaria can be widely diverse, as exemplified by ICEMR research sites in Uganda, Malawi, and Zambia. Comparisons of the impact of malaria control interventions across a range of epidemiological settings are possible using standardized metrics for a given intervention (such as coverage with insecticidal net distribution) and simple or sophisticated measures of transmission intensity (such as force of infection). The range of epidemiological settings encompass areas where *P. vivax* or *P. falciparum* predominate or where mosquito vectors seek blood meals indoors at night or outdoors during early evening. Importantly, epidemiological studies at the ICEMR field sites are integrated with entomological studies, including measurement of the EIR, human biting index, and insecticide resistance, as well as studies of parasite genetic diversity and antimalarial drug resistance. These integrated studies will lead to more detailed and richer understandings of malaria transmission dynamics across the range of epidemiological settings than can be accomplished through studies of only one of these domains. Furthermore, this platform provides the framework for novel studies of malaria epidemiology, including cross-ICEMR investigations of serological responses to an array of parasite antigens and detailed studies of population movement. Epidemiological studies at ICEMR sites will provide a deeper understanding of why specific interventions succeed or fail and will identify surveillance strategies that can be incorporated into standard practice and are sensitive to changes in key malaria indicators.

## Figures and Tables

**Table 1:** Summary of key indicators used for malaria monitoring and evaluation and surveillance

Category	Indicators	Definitions		Primary source of data
Key control interventions	ITN coverage	Proportion of households with at least one ITN		Household surveys
		Proportion of households with at least one ITN for every two people		
		Proportion of proportion of individuals reporting ITN use in previous night		
	IRS coverage	Proportion of households sprayed with IRS within the last 12 months		
		Proportion of population protected by IRS within the last 12 months		
	Case management	Proportion of suspected malaria cases that receive a parasitological test		
		Proportion of confirmed malaria cases that receive first-line therapy		
	IPTp coverage	Proportion of women who received at least three or more doses of IPTp during last pregnancy		
Category	Indicators	Definitions	Comments	Primary source of data
Measures of transmission intensity	EIR	Number of infectious bites received per person per unit time	Widely considered gold standard measure of transmission	Entomology surveys
			Infrequently measured	
			Questions about precision and accuracy	
	Parasite rate	Proportion of population found to carry asexual parasites	Reflection of intensity of transmission, immunity, and effectiveness of treatment	Cross-sectional surveys
			Dependent on age and diagnostic method	
	Force of infection	Number of new infections per person per unit time	Difficult to accurately measure incident events	Cohort studies
			Molecular techniques have been proposed as a means of quantifying new parasite clones acquired	
	Seroprevalence	Proportion of population found to have a positive antibody response to a specific antigen(s)	Have been correlated with EIR	Cross-sectional surveys
			Can provide “snap shot” of historical changes	
			Only a limited number of antigens have been evaluated	
			Lack of standardization in methods used to collect and analyze data	
	Seroconversion rate	Number of new seroconversions per person per unit time. Calculated by fitting a reverse catalytic model to age-specific seroprevalence		
Clinically relevant indicators	Test positivity rate	Proportion of patients with suspected malaria who are tested for infection and test positive	Efficient source of longitudinal data	Health facility-based surveillance
			Dependent on age and diagnostic methods	
	Malaria incidence	Number of confirmed malaria cases per person time of observation	Influenced by case detection methods	Cohort studies or national surveillance systems
			Challenging to accurately measure	
			Subject to misclassification	
			Dependent on age and diagnostic methods	
	Prevalence of anemia	Proportion of population with hemoglobin level below various thresholds	Efficient means of estimating burden of disease	Cross-sectional surveys
			Multifactorial causes of anemia limits use	
			Dependent on age	
	All cause under 5 mortality rate	Probability (expressed as a rate per 1,000 live births) of a child born in a specified year dying before reaching the age of five	May be useful for monitoring temporal trends	National surveillance systems
			Limited by general lack of information on causes of death	
	Malaria case fatality rate	Proportion of malaria cases that result in death	Generally limited to inpatient setting/severe disease	Health facility-based surveillance
			Influenced by source population and quality of care	
			Death may be multifactorial	

EIR = entomological inoculation rate; IPTp = intermittent preventive treatment in pregnancy; IRS = indoor residual spraying; ITN = insecticide-treated bed net.

**Table 2:** Description of ongoing or planned at ICEMR field activities of malaria surveillance, monitoring, and evaluation

ICMER regional centers	Field activities			
	Health facility-based surveillance	Cohort studies	Cross-sectional surveys	Entomology surveys
Malawi*	2 district hospitals (outpatients and inpatients)	1 high transmission site	3 sites	Samples collected from cross-sectional surveys, case–control study and environmental surveys
	1 urban health center (outpatients)	Ages 1–50 years	300 households per site	CDC light traps, household aspiration, larval collections
	1 referral hospital (inpatients)	200 participants	Twice a year	
		Active follow up every month	–	
West Africa	4 community health centers (outpatients)	4 sites (3 rural, 1 urban)	4 sites	4 sites
		All ages	Embedded in the cohort studies	2 weeks before cross-sectional surveys and midseason
		700–1,500 participants per site	Twice a year (beginning and end of transmission season)	30 houses per site
		PCD and active follow-up every month		HLC, PSC
Southern Africa	68 health centers (outpatients)	3 rural sites	3 sites	3 sites
		All ages	150 households per site	Monthly collections
		430 participants	Every other month	175 households per site
		Active follow-up every 2 months		CDC light traps, PSC, larval collections
East Africa	24 health centers (outpatients)	3 sites (2 rural, 1 peri-urban)	3 sites	3 sites
	6 district hospitals (inpatients; children only)	Ages 0.5–10 years and 1 adult per house	200 households per site	Monthly collections
		100 households per site	Once a year	100 houses per site
		PCD and active follow-up every 3 months		CDC light traps (all); HLC, PSC/exit traps (subset)
Amazonia	2 health centers (outpatients)	2 sites	To be designed	6 sites
		Ages ≥ 3 years		HLC, Shannon trap, screen barriers, CDC light traps
		2,000 participants		Monthly collections (rainy season)/bimonthly (dry season)
		Active follow-up every month		
Latin America	4 outpatient clinics	4 sites	5 sites	8 sites with repeated collections **(**8 houses per site)
	3 hospitals (inpatients)	All ages	250–460 households per site	57 sites with single collection (274 houses)
		1,750 participants		CDC light traps, HLC, PSC, larval collections
		Active follow-up every 6 months		
South Asia	2 state and 1 district referral hospitals (outpatients and inpatients)	To be designed	To be designed	4 sites
	6 rural clinics (outpatients)			Weekly collections
				CDC light traps
India*	3 outpatient clinic sites	2 sites	3 sites	2 sites
		Ages 1–70 years	200–300 participants per site	Monthly collections
		200–300 participants per site	Two or three times a year	5–20 houses; 5–18 cattle sheds depending on site
		Active follow-up every 3 months and fortnightly fever surveillance		Aspiration and PSC; larval catches
Southeast Asia	86 outpatient clinics/hospitals	12 sites	12 sites	3 sites
		All ages	∼150 households per site	Collections twice a month
		Total of ∼5,400 participants	3–4 times a year	30–60 houses per site
		Weekly household visits		Light traps
Southwestern Pacific	5 clinic sites (outpatients)	3 sites	3 sites	5 sites
		Ages 0.5–12 years	2,500 participants per site	Landing catch and barrier screen methods
		450–800 participants per site	Once a year	
		Active follow up every month		

CDC = Centers for Disease Control and Prevention; HLC = human landing catches; ICEMR = International Centers of Excellence for Malaria Research; PCD = passive case detection; PSC = pyrethrum spray catches.

* Miscellaneous work includes case–control study of urban malaria (Malawi), reactive case detection (India).

**Table 3:** Key malaria metrics at ICEMR field sites

ICMER	Country	Site	Predominant parasites	Predominant vectors	Season	EIR	Parasite prevalence	ITN coverage (%)	IRS coverage (%)
Africa									
East Africa	Uganda	Jinja	*Plasmodium falciparum*	*Anopheles arabiensis*	Perennial	3	16% (2–10 years)	58	3
				*Anopheles gambiae* s.s.					
	Uganda	Kanungu	*P. falciparum*	*An. gambiae* s.s.	Perennial	30	18% (2–10 years)	51	0
	Uganda	Tororo	*P. falciparum*	*An. gambiae* s.s.	Perennial	310	60% (2–10 years)	79	0
Malawi	Malawi	Blantyre	*P. falciparum*	*Anopheles funestus*	Single	NA	9% (all ages)	84	2
				*An. arabiensis*					
	Malawi	Thyolo	*P. falciparum*	*An. funestus*	Single	NA	14% (all ages)	85	0
				*An. arabiensis*					
	Malawi	Chikwawa	*P. falciparum*	*An. funestus*	Perennial	100	30% (all ages)	93	73
				*An. arabiensis*					
				*An. gambiae* s.s					
Southern Africa	Zambia	Nchelenge	*P. falciparum*	*An. funestus*	Perennial	140	50% (all ages)	62	28
				*An. gambiae*					
	Zambia	Choma	*P. falciparum*	*An. arabiensis*	Single	< 1	1% (all ages)	83	0
	Zimbabwe	Mutasa	*P. falciparum*	*An. funestus*	Single	10	10% (all ages)	59	57
				*An. gambiae*					
West Africa	Gambia	Gambissara	*P. falciparum*	*An. arabiensis*	Single	25	8% (all ages)	85	95
				*An. gambiae*					
	Senegal	Thiès	*P. falciparum*	*An. funestus*	Single	1	1% (all ages)	20	0
				*An. arabiensis*					
				*An. gambiae*					
	Mali	Dangassa	*P. falciparum*	*An. gambiae*	Single	50	48% (all ages)	30	0
				*An. arabiensis*					
				*An. funestus*					
	Mali	Dioro	*P. falciparum*	*An. gambiae*	Single	5	24% (all ages)	95%	0
				*An. arabiensis*					
				*An. funestus*					
South America									
Amazonia	Peru	Loreto	*Plasmodium vivax*	*Anopheles darlingi*	Perennial	5	9% (all ages)	70	55
			*P. falciparum*						
Latin America	Colombia	Tierralta	*P. vivax*	*Anopheles nuneztovari*	Perennial	3	6% (all ages)	88	0
			*P. falciparum*	*An. darling*					
				*Anopheles albimanus*					
	Colombia	Buenaventura	*P. vivax*	*An. nuneztovari*	Perennial	< 1	5% (all ages)	95	0
			*P. falciparum*	*Anopheles pseudopunctipennis*					
				*An. albimanus*					
	Colombia	Tumaco	*P. vivax*	*An. albimanus*	Perennial	3	6% (all ages)	97	0
			*P. falciparum*	*An. calderoni*					
	Peru	Sullana	*P. vivax*	*An. albimanus*	Single	< 1	1% (all ages)	44	80
			*P. falciparum*						
Asia and South Pacific									
India	India	Chennai	*P. vivax*	*An. stephensi*	Perennial	NA	5.7% *P. vivax*	0	0
			*P. falciparum*				0.4% *P. falciparum*		
	India	Raurkela	*P. vivax*	*An. fluviatilis*	Perennial	7.3–127	1.5% *P. vivax*	60	5
			*P. falciparum*	*An. culicifacies*			5% *P. falciparum*		
			*P. malariae*						
	India	Nadiad	*P. vivax*	*An. culicifacies*	Single	0.05–0.21	5.7% *P. vivax*	1	9
			*P. falciparum*				2.4% *P. falciparum*		
South Asia	India	Goa	*P. falciparum*	*An. stephensi*	Perennial	2.5	0.4%	0	0
			*P. vivax*						
	India	Wardha	*P. falciparum*	*An. culicifacies*	Perennial	NA	0.2%	0	0
			*P. vivax*						
	India	Ranchi	*P. falciparum*	*An. culicifacies*	Perennial	NA	1.4%	NA	NA
			*P. vivax*	*An. fluviatilis*					
	India	Assam	*P. falciparum*	*An. baimaii*	Perennial	0.4	6% (all ages)	79	40
			*P. vivax*	*An. minimus*		0.1			
			*Plasmodium ovale*						
			*Plasmodium malariae*						
Southeast Asia	China	Ying Jiang	*P. vivax*	*An. minimus*	Perennial	0.1	NA	80	100
			*P. falciparum*	*An. maculatus*					
				*An. sinensis*					
	Myanmar	Laiza	*P. vivax*	*An. minimus*	Perennial	0.4	1% (all ages)	96	100
			*P. falciparum*	*An. maculatus*					
				*An. culicifacies*					
	Thailand	Tha Song Yang	*P. vivax*	*An. minimus*	Perennial	0.3	0.3% (all ages)	90	72
			*P. falciparum*	*An. maculatus*					
				*An. annularis*					
Southwest Pacific	PNG	East Sepik	*P. falciparum*	*An. punctulatus*	Perennial	14	7% *P. falciparum*	96	0
			*P. vivax*	*An. farauti*			4% *P. vivax*		
			*P. ovale*	*An. koliensis*			1% *P. malariae*		
			*P. malariae*				1% *P. ovale*		
							(all ages)		
	PNG	Madang	*P. falciparum*	*An. punctulatus*	Perennial	40	19% *P. falciparum*	96	0
			*P. vivax*	*An. farauti*			13% *P. vivax*		
			*P. ovale*	*An. koliensis*			–		
			*P. malariae*				–		
	Solomon Islands	Central Province	*P. vivax*	*An. farauti*	Perennial	40	14% *P. vivax* (1–80 years)	90	81
			*P. falciparum*						
	Solomon Islands	Western Province	*P. vivax*	*An. farauti*	Perennial	NA	NA	95	NA

EIR = entomological inoculation rate (number of infectious bites per year); ICEMR = International Centers of Excellence for Malaria Research; IRS = indoor residual spraying; ITN = insecticide-treated bed net; ITN coverage = proportion of households with at least two ITNs; IRS coverage = proportion of households sprayed within insecticide within the past year; NA = not available; Parasite prevalence = proportion of individuals in specified age groups positive for malaria by RDT or microscopy; PNG = Papua New Guinea; PPY = person per year.

## References

[1] Hay SI, Smith DL, Snow RW (2008). Measuring malaria endemicity from intense to interrupted transmission. Lancet Infect Dis.

[2] Mendis K, Rietveld A, Warsame M, Bosman A, Greenwood B, Wernsdorfer WH (2009). From malaria control to eradication: the WHO perspective. Trop Med Int Health.

[3] Gillies MI, Wernsdorfer WH, McGregor I (1988). Malaria: Principles and Practice of Malariology.

[4] Tusting LS, Bousema T, Smith DL, Drakeley C (2014). Measuring changes in *Plasmodium falciparum* transmission: precision, accuracy and costs of metrics. Adv Parasitol.

[5] Nabarro DN, Tayler EM (1998). The “roll back malaria” campaign. Science.

[6] Report WM (2012). World Health Organization.

[7] Noor AM, Kinyoki DK, Mundia CW, Kabaria CW, Mutua JW, Alegana VA, Fall IS, Snow RW (2014). The changing risk of *Plasmodium falciparum* malaria infection in Africa: 2000–10: a spatial and temporal analysis of transmission intensity. Lancet.

[8] Alonso PL, Tanner M (2013). Public health challenges and prospects for malaria control and elimination. Nat Med.

[9] Bousema T, Okell L, Felger I, Drakeley C (2014). Asymptomatic malaria infections: detectability, transmissibility and public health relevance. Nat Rev Microbiol.

[10] Kelly GC, Hale E, Donald W, Batarii W, Bugoro H, Nausien J, Smale J, Palmer K, Bobogare A, Taleo G, Vallely A, Tanner M, Vestergaard LS, Clements AC (2013). A high-resolution geospatial surveillance-response system for malaria elimination in Solomon Islands and Vanuatu. Malar J.

[11] Mueller I, Schoepflin S, Smith TA, Benton KL, Bretscher MT, Lin E, Kiniboro B, Zimmerman PA, Speed TP, Siba P, Felger I (2012). Force of infection is key to understanding the epidemiology of *Plasmodium falciparum* malaria in Papua New Guinean children. Proc Natl Acad Sci USA.

[12] Report WM (2013). World Health Organization.

[13] Molineaux L, Gramiccia G (1980). The Garki Project: Research on the Epidemiology and Control of Malaria in the Sudan Savanna of West Africa.

[14] Hay SI, Snow RW (2006). The malaria Atlas Project: developing global maps of malaria risk. PLoS Med.

[15] Greenwood BM (1989). The microepidemiology of malaria and its importance to malaria control. Trans R Soc Trop Med Hyg.

